# Effects of Deep Brain Stimulation on Eye Movements and Vestibular Function

**DOI:** 10.3389/fneur.2018.00444

**Published:** 2018-06-12

**Authors:** Aasef G. Shaikh, Chrystalina Antoniades, James Fitzgerald, Fatema F. Ghasia

**Affiliations:** ^1^Department of Neurology, University Hospitals, Case Western Reserve University, Cleveland, OH, United States; ^2^Daroff-Dell'Osso Ocular Motility Laboratory, Cleveland VA Medical Center, Cleveland, OH, United States; ^3^NeuroMetrology Lab, Nuffield Department of Clinical Neurosciences, University of Oxford, Oxford, United Kingdom; ^4^Nuffield Department of Surgical Sciences, University of Oxford, Oxford, United Kingdom; ^5^Cole Eye Institute, Cleveland Clinic, Cleveland, OH, United States

**Keywords:** Parkinson's disease, tremor, dystonia, neuromodulation, saccade, pursuit, gaze holding

## Abstract

Discovery of inter-latching circuits in the basal ganglia and invention of deep brain stimulation (DBS) for their modulation is a breakthrough in basic and clinical neuroscience. The DBS not only changes the quality of life of hundreds of thousands of people with intractable movement disorders, but it also offers a unique opportunity to understand how the basal ganglia interacts with other neural structures. An attractive yet less explored area is the study of DBS on eye movements and vestibular function. From the clinical perspective such studies provide valuable guidance in efficient programming of stimulation profile leading to optimal motor outcome. From the scientific standpoint such studies offer the ability to assess the outcomes of basal ganglia stimulation on eye movement behavior in cognitive as well as in motor domains. Understanding the influence of DBS on ocular motor function also leads to analogies to interpret its effects on complex appendicular and axial motor function. This review focuses on the influence of globus pallidus, subthalamic nucleus, and thalamus DBS on ocular motor and vestibular functions. The anatomy and physiology of basal ganglia, pertinent to the principles of DBS and ocular motility, is discussed. Interpretation of the effects of electrical stimulation of the basal ganglia in Parkinson's disease requires understanding of baseline ocular motor function in the diseased brain. Therefore we have also discussed the baseline ocular motor deficits in these patients and how the DBS changes such functions.

## Introduction

Deep brain stimulation (DBS) is the standard of care in treatment of movement disorders including Parkinson's disease (PD), essential tremor, and dystonia. In addition to the compelling clinical benefits seen in the over 100,000 movement disorders patients treated worldwide, DBS also offers an opportunity to study the effects of electrical stimulation of the basal ganglia on the physiology of motor, sensory, or cognitive systems. The focus of this review is to discuss the effects of DBS of globus pallidus internus (GPi), subthalamic nucleus (STN), and thalamus on ocular motor and vestibular functions. While interpreting the effects of DBS on any aspect of physiology, it is critical to appreciate that such surgery is by definition performed on diseased brains and the effects of stimulation are conflated with the effects of the condition being treated. Nevertheless with a good understanding of the baseline eye movement abnormalities in the patient population concerned, and suitable healthy controls where needed, it is possible to gain insights into normal physiology, disease pathophysiology, and how DBS affects them (1).

## How does DBS work?

Although DBS can dramatically improve the motor symptoms of PD, essential tremor, and dystonia, its physiological mechanism of action remains unclear. Because stimulation and lesional surgery at the same sites produce similar beneficial effects in PD, it was previously believed that DBS produced a “physiological ablation” of the target. This is consistent with a simple physiological model of the basal ganglia and PD, where symptoms are due to increased activity of the STN and GPi. It was, therefore, hypothesized that DBS improves clinical symptoms by suppressing the *outflow* of the basal ganglia.

This simple view is no longer tenable, but the mechanism of action of DBS remains poorly understood with several possible explanations (2–4). Stimulation almost certainly causes neuronal excitation, most likely of axons, which have lower firing thresholds than neuronal somata. The consequence of this is likely not to be simply the up-regulation or down-regulation of one or more nuclei, but rather the disruption of some form of pathological network activity. Contemporary studies suggest hyper-synchronization of spontaneous neural activity as a cause of tremor and rigidity. It follows that de-synchronization of such activity might resolve these symptoms. De-synchronization is possible by lesioning the parts of hyper-synchronized circuit (e.g., pallidotomy or thalamotomy), or electrically stimulating (thereby suppressing) the circuit (e.g., DBS) (5, 6). Another possible type of pathological activity is “neural noise” due to excessive random firing, as is seen in the medium spiny neurons of the striatum in PD. Such noise can interfere with information flow within the basal ganglia and it has been suggested that pallidal DBS might act to dampen the noise (7).

## Applied anatomy, physiology, and pathophysiology of basal ganglia in ocular motor control

**Figure 1:**
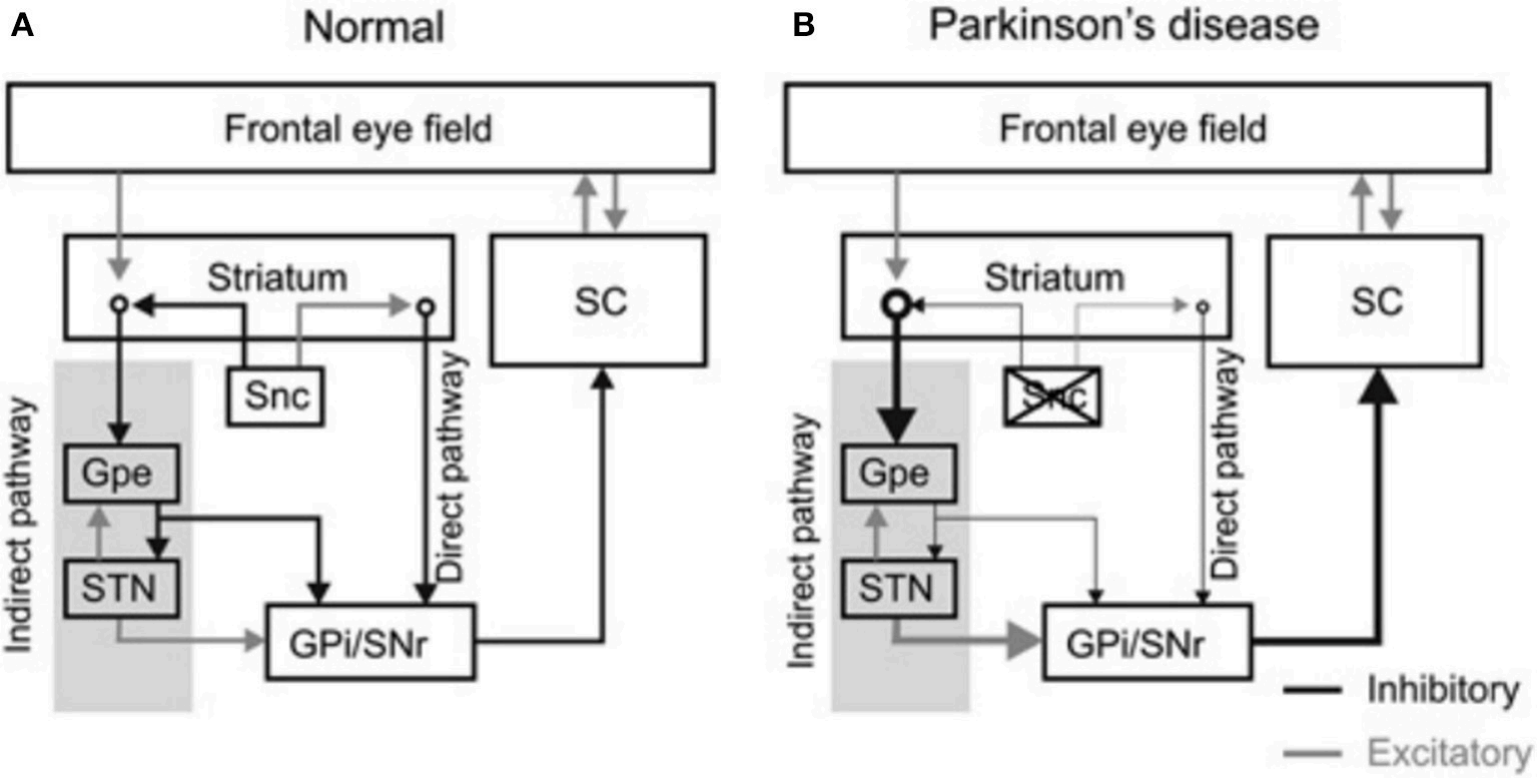
Diagram summarizes organization of the basal ganglia nuclei and their projections. **(A)** The substantia nigra pars compacta (Snc) sends excitatory and inhibitory projections to the striatum. Latter subsequently projects to the basal ganglia outflow nuclei—the globus pallidum internus (GPi) and substantia nigra pars reticulata (SNr) via, parallel, direct and indirect pathways. The direct pathway neurons in the striatum receive excitatory projections from the SNc and sends direct inhibitory projections to GPi and SNr, while inhibitory projections from SNc are sent to the striatal indirect pathway. The indirect pathway, via globus pallidum externus (GPe) and subthalamic nucleus (STN), influences the SNr and GPi. As a result of these connections the dopaminergic neuronal degeneration, as seen in Parkinson's disease, leads to disinhibition of GPi and SNr. Such dysinhibtion leads to inappropriate inhibition of the superior colliculus (SC) (relative thickness of the arrows represents the strength of projection **(B)**. [Figure and corresponding legend modified with permission from (10)].

The frontal eye field (FEF), supplementary eye field (SEF), dorsolateral prefrontal cortex (DLPFC), and the parietal eye fields project to the basal ganglia, which then relay this input to the superior colliculus (8, 9). The cortical areas project to the caudate nucleus which sends direct inhibitory fibers to the substantia nigra pars reticulata (SNr) and an indirect projection to the STN via the external segment of the globus pallidus (Figure 1A) [The figure modified with permission from (10)]. The SNr maintains tonic GABAergic inhibition on the superior colliculus (11, 12); timely and transient cessation of such inhibitory control leads to timely saccade initiation (9, 13, 14). Lesions of the caudate nucleus decrease the velocity and the amplitude of saccades (15). Pharmacological inhibition of the SNr after muscimol injection results in saccadic intrusions and contralaterally directed spontaneous saccades (16). Electrical stimulation of the SNr results in reduced latency and hypometric memory or visually guided saccades (17). In pathological states such as PD, the superior colliculus remains in an inhibited state due to hyperactivity of the SNr (See thick arrow Figure 1B) [the figure modified with permission from (10)].

## Eye movement abnormalities in PD

**Figure 2:**
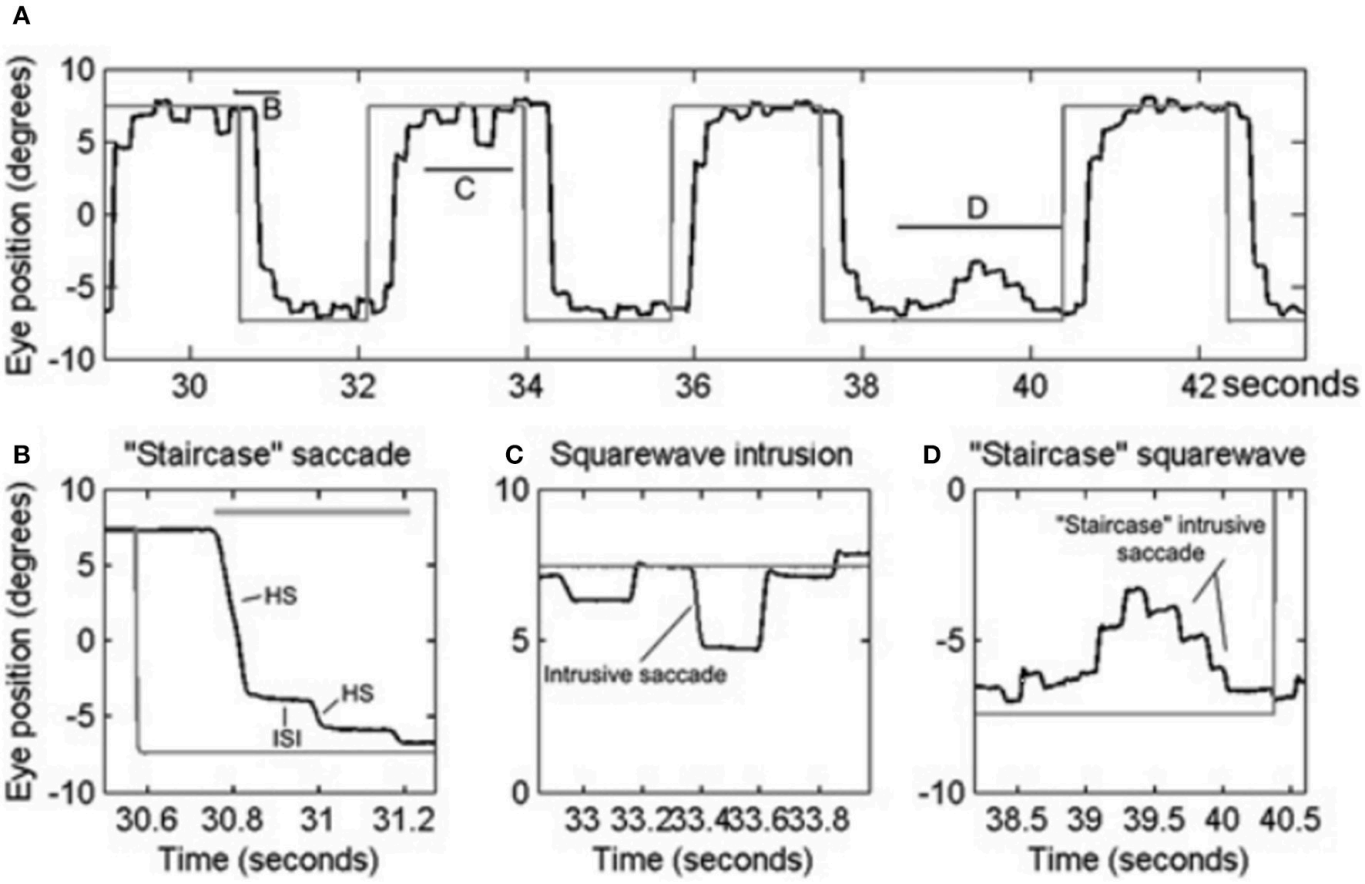
Example of saccadic and fixation deficit in one patient with early Parkinson's disease. Horizontal eye positions are depicted with black trace; the gray trace represents the visual target position. **(A)** Saccade and fixation abnormality is summarized from one patient. There is hypometria of initial saccade and it is then followed by several hypometric corrective saccades (HS). Latter brings the gaze to the target. Horizontal lines in **(A)** depict the portion of the eye movement trace that is temporally zoomed in **(B–D)**. Thick, horizontal, gray line is the target acquisition time. **(C)** Depicts an example of square-wave jerk (SWJ), while **(D)** shows “staircase” square-wave jerk. ISI, inter-saccadic interval. [Figure and legend reproduced with permission from (10)].

PD is a common neurodegenerative disorder with a complex and variable mixture of clinical features including slowness of movements (bradykinesia), resting tremor, shuffling gait, mask-like facies, and increased muscle tone. A wide range of eye movement abnormalities have been reported in PD, affecting smooth pursuit, vergence, fixation, saccades, and gaze holding (10, 18–23). Abnormalities in smooth pursuit eye movements include a reduction in the eye velocity relative to target movement, i.e., decreased smooth pursuit gain. The reduction in gain worsens as the disease progresses (21). Insufficiency of both convergence and divergence has been described (24–26). A reduction in smooth pursuit gain, mild restriction in upgaze (27), and convergence insufficiency is also seen in elderly who are otherwise healthy, but is more marked in PD. Subjects with PD have saccadic intrusions during attempted steady gaze fixation, followed by return; these movements are called square-wave jerks (10, 20, 28). While square-waves [see Figures 2A,C; the figure reproduced with permission from 10] are often notable in healthy individuals (29), their hypometric characteristic followed by a catch up saccade, just like hypometric visually guided saccades (see Figure 2B), (“staircase square-waves”) is a unique feature of PD [see Figures 2A,D; the figure reproduced with permission from (10)].

Several studies have shown abnormalities in visually guided saccades in subjects with PD. Visually guided prosaccades (saccades toward a novel target) show increased latency (30–34), which progresses over time. Analyzing the distribution of latencies over multiple trials can help in the differential diagnosis of PD and other parkinsonian syndromes (35). Surprisingly, treatment with levodopa, despite alleviating motor symptoms, further increases latency (36). Patients with PD also often have difficulty in inhibiting the reflexive prosaccadic response or initiating a voluntary response in the opposite direction during the antisaccade task. This leads to a higher than normal antisaccadic error rate (AER) (37, 38).

Saccades in parkinsonian patients show an increased prevalence of hypometria, compared to healthy controls (10, 22). Asymmetry of hypometria can be seen in subjects with asymmetric PD, the saccades being more hypometric on the more symptomatic side (39). Series of hypometric saccades that ultimately leads to shift the gaze to its target appears like a “staircase” (40, 41). While staircases increase the time taken to reach the target of interest, this does not imply reduction in the saccade velocity (10). Although there is increased variability in the peak velocity, only the subjects with advanced PD have saccade slowing (21, 28, 42). Correlation between eye movement abnormalities and freezing of gait has also been observed in PD (20, 43, 44). PD patients with freezing of gait have increased antisaccade latency and variability in the saccade velocity and accuracy (44).

## Effects of DBS on saccades

DBS is known to influence some saccade abnormalities; for example, prosaccade latency is reduced by the STN or GPi DBS (45–47). DBS is unique in doing this: both levodopa treatment and lesional surgery do the opposite, despite the fact that both of these treatments produce symptomatic improvement. This is further evidence that the physiological mechanism of action of DBS is not simply a physiological ablation. The STN not only facilitates saccades, but also plays a critical role in their planning. Recordings of local field potentials from STN DBS electrodes show event related desynchronizations in the beta band immediately prior to saccades (48) that are qualitatively similar to those seen in motor planning of limb movement.

It has been proposed that the basal ganglia comprise a network that is capable of performing Bayesian statistics for movement selection (49, 50). In this scheme the STN feedback assures that increase in probability of one action is linked with reduction in the probability of other available options (51). Studies examining the effects of STN DBS on saccade generation have provided evidence for this concept. PD patients were asked to make horizontal saccades to a target that appeared either to the left or to the right. The investigators periodically changed the probability of the target appearing in each location and found that saccadic latencies shortened as the target location became more probable, or vice versa. When DBS was turned on, the latency for the more probable target location remained shortened, but the reaction time for less probable locations failed to increase. Disrupting the STN output interfered with the normalized representation of prior probabilities (7, 52).

Both STN and GPi DBS improve prosaccadic latencies. Interestingly GPi stimulation (but not STN stimulation) also reduces the AER (53). While changes in prosaccade latency could be accounted for exclusively by effects within the basal ganglia, the antisaccade task involves higher level functions including inhibition of a reflexive prosaccade and the subsequent volitional generation of a saccade in the opposite direction, both of which are functions of the prefrontal cortex. The results of this study imply that GPi DBS can improve deficits in higher control of lower motor functions (53).

## Effects of DBS on smooth pursuit eye movements

Video-oculography in 34 patients with PD revealed decreased smooth pursuit eye movement velocity compared to the velocity of a pursued target (i.e., reduced smooth pursuit gain) (54). There is conflicting literature on the effects of DBS on smooth pursuit eye movements. In one study including 14 STN DBS patients, stimulation did not affect pursuit eye movements (54), while in another study of 9 DBS patients both smooth pursuit velocity and accuracy were significantly increased with stimulation (55). Globus pallidus, via thalamic relay, projects to the smooth pursuit areas of the frontal eye fields (56, 57). It is therefore possible that basal ganglia outflow modulates the extended cortical network responsible for smooth pursuit (55).

## Effects of DBS on gaze holding

Square-wave jerks are defined by spontaneous intrusive saccades that takes the gaze away from the target followed by a return saccade within 200 ms intersaccadic interval (58). Although square-wave jerks are common in atypical parkinsonism such as multiple system atrophy and progressive supranuclear palsy; approximately 20% of patients with early idiopathic PD (10, 20, 23). Square-wave jerks in early PD are often interrupted, giving the appearance of a “staircase” [see Figures 2A,D; the figure reproduced with permission from (10)]. We hypothesized that enhanced inhibition of the superior colliculus in the parkinsonian state may lead to reduced inhibition of presaccadic activity FEF activity leading to its phasic increase and subsequently the square-wave jerk (10). Interruptions of the large intrusive saccades comprising the square-wave jerk, due to phasic SNr inhibition, leads to its “staircase” pattern [see Figures 2A,D] (10) [The figure reproduced with permission from (10)].

Pallidotomy increases the frequency of square wave jerks in patients with PD (59). As an explanation, it was proposed that the disinhibition of the ascending thalamocortical loops can reactivate the prefrontal cortex creating imbalance in the activity of the saccade-related prefrontal structures such as the frontal eye fields and supplementary motor eye fields (59). In a study examining the effects of STN DBS on square-wave jerks, the authors found reduction in the frequency of intrusive saccades after bilateral DBS (60).

## Single-unit activity in human basal ganglia and eye movements

The functional changes in the ocular motor system resulting from pallidal and subthalamic DBS prompt a fundamental question—is the STN or pallidum directly related to ocular motor control, i.e., do eye movement sensitive neurons exist in the STN or pallidum, or does the stimulation indirectly affect downstream ocular motor sensitive areas such as the SNr or superior colliculus to manifest its effects? Intraoperative microelectrode recordings and single channel electrooculography in 19 PD patients addressed this question. Intraoperatively, while simultaneously sampling single unit activity and oculography, the patients were asked to view a series of colored pictures and perform a visually guided saccade task (61). About one-fifth of neurons isolated from the SNr, globus pallidus, and STN had direct eye movement sensitivity (61).

## DBS and eyelid motor control

DBS in a rodent model of parkinsonism was recently used to study blink hyper-reflexia, impaired reflex blink plasticity, and reduced spontaneous blink rate (62). A hyper-synchronized beta-band in the basal ganglia output was found to be associated with such blink abnormalities. High-frequency DBS of the STN affected blink hyper-reflexia and blink reflex plasticity; however there were no effects on the abnormal rate and rhythm of the spontaneous blinks (62). It is possible that that the stimulated area of STN was away from that controlling spontaneous blink rate and pattern. It is also possible that DBS parameters used in the experiments were not suitable to change the spontaneous blink abnormality. Finally it can also be speculated that DBS does not affect the discharge pattern of the basal ganglia activity, but it simply reduces the neural response gain. A clinical study showed that pallidal DBS in humans with tardive as well as axial dystonia improves forced eyelid closures (blepharospasm) (63, 64).

## Effects of DBS on the vestibular system

The central vestibular system facilitates stable gaze holding, the motion perception, and orientation. The brainstem, under the cerebellar guidance, modulates these functions. In support of this theory, contemporary investigations identified the disorders of human brain where motion perception is selectively affected (65–76). The discovery of non-eye movement sensitive brainstem and cerebellar vestibular neurons further supported this concept (65, 67–69, 77, 78). The non-eye movement sensitive brainstem and cerebellar neurons may have specialized role in encoding a central representation of the gravitational force (65, 67, 70), heading direction (66, 70, 79). The cerebellum sends direct projections, via the vestibulo-thalamic track (fibers that are adjacent to the medial lamniscus but medial and then dorso-medial to the STN), to the ventro-posterior and ventro-lateral thalamus (77, 78, 80). It is therefore predicted that inadvertent stimulation of the vestibulo-thalamic projections can lead to abnormal interpretation of gravitational force. A recent study investigated the effects of DBS of the nucleus ventralis intermedius of the thalamus revealed change in sense of visual verticality (subjective visual vertical) when electrical stimulation was turned on. The patients felt a tilt at 1.4 ± 0.4 degrees on the contraversive side when the stimulator was on; in contrast when stimulator was turned off, the contraversive tilt was 4.4 ± 3.0 degrees (81).

**Figure 3:**
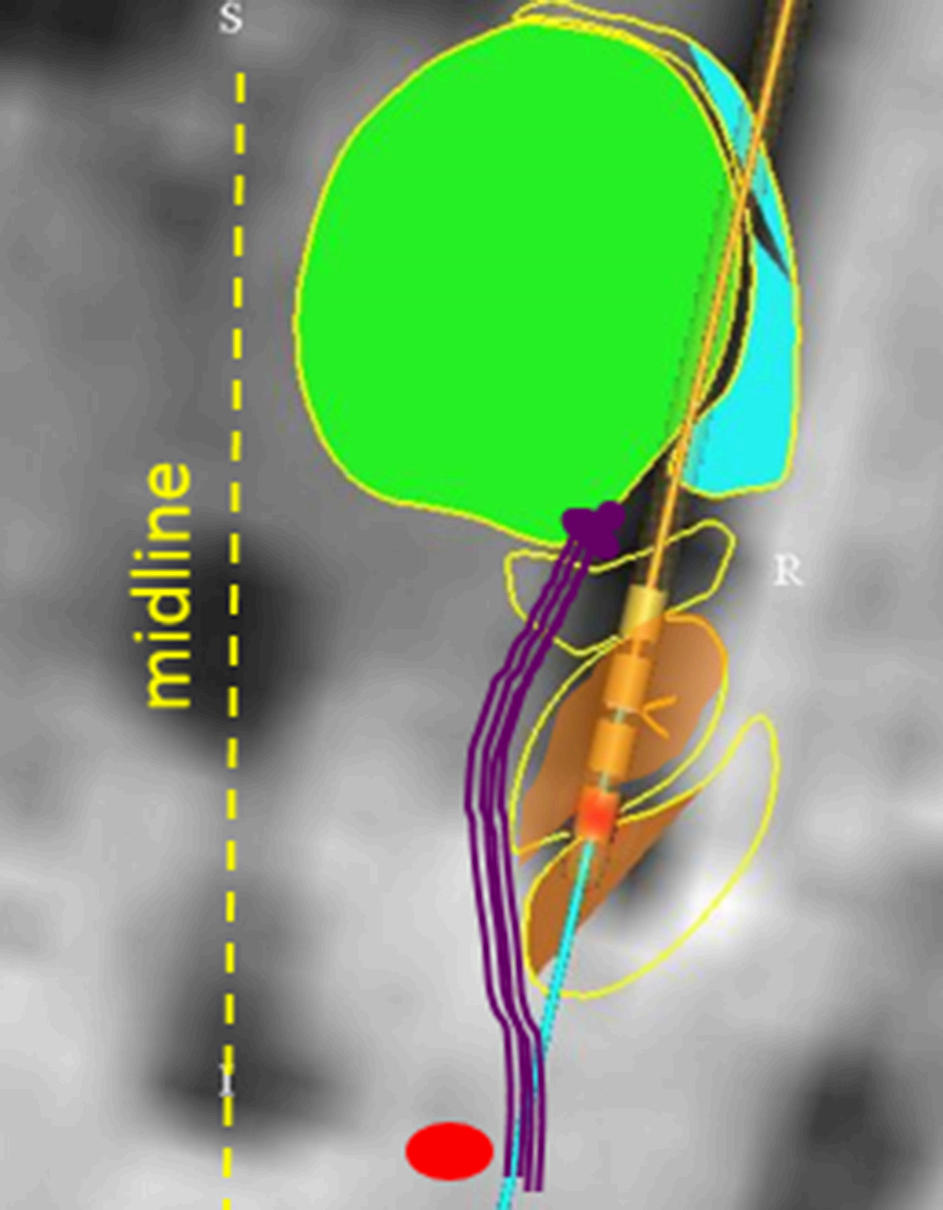
Anatomical model that reconstructed the basal ganglia subnuclei and coordinates of DBS electrode placed in STN. The green area is the model fitted to the thalamus, orange area (yellow arrow) is fitted to STN. The red circle depicts red nucleus. Vestibulo-thalamic fibers (schematized with purple lines) are medial to the STN before they course on the medio-dorsal border of STN to enter the thalamus. DBS electrode leads are depicted with four cylinder shapes (black arrow); red colored cylinder is contact #0 on the implanted lead (Medtronic 3389). [Figure and corresponding legend modified with permission from (83)].

Inadvertent stimulation of medial longitudinal fasciculus or the interstitial nucleus of Cajal can lead to isolated ipsilateral head tilt (82). It is also possible that altered perception of visual vertical (orientation of self in relation to the gravity), due to inadvertent stimulation of the vestibulo-thalamic fibers, have led to reactive head tilt. Recent study investigated the effects of STN stimulation through the medial and caudal DBS electrode contact in five PD subjects (83). Imaging and electrode location from one subject is depicted in Figure 3 [The figure modified with permission from (83)]. Perception of rotational motion in the plane of horizontal semicircular canal was most commonly reported by these patients, one patient also felt as if she was riding a swing. Latter form of complex perception could be due to the combined stimulation of the vestibulo-thalamic fibers conducting vertical semicircular canals and otolith derived signals, i.e., combination of pitch and fore-aft motion respectively. These serendipitous findings brought new insight into counter-intuitively implementing DBS for the treatment of vertigo and imbalance due to abnormal motion perception.

## Conclusions

Although DBS is predominantly viewed as modulating appendicular and axial motor function, tremor, and dystonia, it also has a substantial influence on ocular motor and vestibular function. Clinically, knowledge of how these systems may be affected by stimulation, together with knowledge of the anatomical locations of motor structures relative to ocular motor regions, is critical for safe and effective programming of stimulation parameters. The underlying mechanism of action of DBS is unclear, and because the neurophysiology of eye movements is better understood, the studies of DBS on ocular motor function may offer analogies that help to interpret its effects on appendicular and axial motor function. We also acknowledge that on several occasions the DBS studies have resulted in inconsistent effects on ocular motor function. Such incoherencies could result from institutional (minor) variabilities in DBS electrode placements as well as the variabilities in tested therapeutic electrode contacts. The next generation of studies correlating the outcome of DBS on ocular motor parameters and electrical tissue activation models are desperately needed.

## Author contributions

AS conceptualized the manuscript, authored manuscript. CA, JF, and FG edited manuscript.

## Conflict of interest statement

The authors declare that the research was conducted in the absence of any commercial or financial relationships that could be construed as a potential conflict of interest.
